# Evaluating Maternal Accuracy in Smartphone‐Based Tele‐Dentistry for Early Childhood Caries Detection

**DOI:** 10.1002/cre2.70231

**Published:** 2025-10-03

**Authors:** Parastoo Iranparvar, Zahra Ghorbani, Sajjad Zandieh, Serlie Hartoonian, Mohsen Shirazi

**Affiliations:** ^1^ Department of Pediatric Dentistry, Shahid Beheshti University of Medical Sciences School of Dentistry Tehran Iran; ^2^ Department of Community Oral Health, Shahid Beheshti University of Medical Sciences School of Dentistry Tehran Iran; ^3^ The Iranian Academy of Medical Sciences Tehran Iran; ^4^ Department of Orthodontics, School of Dentistry Tehran University of Medical Sciences Tehran Iran

**Keywords:** digital health, early childhood caries, maternal screening, mobile health, pediatric dentistry, preventive oral care, tele‐dentistry

## Abstract

**Background:**

Early childhood caries (ECC) is a public health challenge, leading to long‐term dental problems and costs. Limited access to preventive care underscores the need for innovative screening methods. Tele‐dentistry, using smartphone‐based imaging, offers a scalable solution for ECC detection and intervention.

**Objective:**

This study evaluates the accuracy of maternal smartphone‐based ECC assessments compared to professional evaluations as a reliable screening tool.

**Methods:**

A cross‐sectional‐study recruited 114 mother–child pairs from primary healthcare centers in Tehran province, Iran. Mothers received online training and conducted visual and smartphone‐based caries assessments. Two blinded pediatric dentists remotely reviewed images, with findings compared to in‐person dental examinations. Sensitivity, specificity, and intraclass correlation coefficients (ICCs) were calculated.

**Results:**

The mean child age was 4.8 ± 0.9 years with 56.1% boys and 43.9% girls. Mothers had a mean age of 33.2 ± 5.6 years with 55% having a high school diploma or higher. Maternal assessments identified caries in 49/58 cases (84.5%), while pediatric dentists detected 51/58 cases (87.9%). Maternal assessments showed 100% sensitivity and specificity, with strong agreement with professional evaluations (ICC = 0.909–0.988, *p* < 0.0001). The three methods demonstrated near‐perfect concordance (ICC = 0.978, *p* < 0.0001).

**Conclusion:**

Maternal‐assisted tele‐dentistry is a reliable, accessible screening method for ECC, improving early intervention, especially in low resources settings and underserved communities.

## Summary

- Innovative Home‐Based Screening—Validating maternal‐assisted smartphone screening, demonstrating its potential for early caries detection.
- Empowering Parents in Preventive Care—Trained mothers play an active role in oral health, reinforcing preventive behaviors and reducing unnecessary dental visits.
- Reliable and Scalable Solution—High ICC values and 100% sensitivity/specificity confirm its accuracy, supporting tele‐dentistry as a cost‐effective, sustainable screening tool.

## 1 Introduction

Early childhood caries (ECC) is a major global public health concern, affecting millions of children, with significant regional disparities. Recent estimates indicate that 53% of children aged 1–5 years worldwide are affected, with prevalence rates reaching 88.8% in certain low‐ and middle‐income regions such as the Middle East and North Africa (MENA) (Javadzadeh et al. 2023; Elamin et al. 2021). Some countries report particularly high ECC rates, including 73% in Saudi Arabia, 83% in the United Arab Emirates, and 89% in Qatar (Alkhtib and Mohamed 2023). Limited access to preventive dental care, low maternal education, and high sugar consumption contribute to these high rates (Ghorbani et al. 2018). Given these challenges, innovative screening approaches like tele‐dentistry have emerged as promising tools to enhance early caries detection and intervention.

Tele‐dentistry, particularly through smartphone‐based imaging, has demonstrated strong potential for remote caries screening (Estai et al. 2020, 2022). Studies indicate that sensitivity exceeds 80% and specificity surpasses 90%, with diagnostic agreement comparable to in‐person exams (Estai et al. 2022). However, prior research has largely focused on school‐based screenings or professional image capture, rather than home‐based assessments conducted by parents (Ghai 2020; Mohebbi et al. 2017). Expanding tele‐dentistry to include parental involvement could enhance access to early screening, reduce diagnostic delays, and minimize unnecessary dental visits, particularly in underserved communities.

Incorporating caregivers into tele‐dentistry is a novel approach, as existing models have primarily relied on dental professionals to capture and interpret images (Ghai 2020; Mohebbi et al. 2017). Training and empowering mothers to perform initial caries screenings at home could bridge gaps in pediatric dental care where professional services are limited or delayed. Prior studies have demonstrated that trained caregivers can perform preventive dental tasks with accuracy comparable to professionals. Mohebbi et al. (2017) found that mothers trained in fluoride varnish application achieved results nearly identical to dental students, with 96% feeling confident in repeating the procedure after 6 months (Mohebbi et al. 2017). These findings suggest that parental training could be extended beyond preventive care to include smartphone‐based caries detection, improving access to early diagnosis and intervention.

This study evaluates the accuracy of maternal smartphone‐based ECC assessments compared to professional evaluations, incorporating structured parental training. We hypothesize that trained mothers will demonstrate high diagnostic accuracy, with strong agreement with professional assessments. To enhance rigor, we use intraclass correlation coefficients (ICCs) to compare maternal, remote dentist, and in‐person evaluations, addressing a key research gap in tele‐dentistry. Unlike previous studies focused on school‐based screenings or professional‐led image capture, this study assessed maternal‐assisted smartphone screening for ECC. If validated, this model could offer a scalable, cost‐effective approach to integrating oral health screening into primary care, improving early detection, and reducing disparities.

## 2 Methods

This cross‐sectional observational study was conducted from 2021 to 2022 to evaluate the accuracy of maternal participation in tele‐dentistry for ECC detection using smartphone‐based dental imaging. Mother–child pairs were recruited from those having an existing electronic record in primary healthcare (PHC) centers and dental clinics affiliated with the Shahid Beheshti University of Medical Sciences, School of Dentistry, Tehran, Iran. Inclusion criteria encompassed children aged 3–6 years with at least one primary tooth and mothers who owned smartphones and provided informed consent. Exclusion criteria included children with severe dental anomalies or significant developmental disabilities that could impede cooperation during the study procedures. The study protocol received ethical approval from the Institutional Review Board (IRB) of the Iranian Academy of Medical Sciences and Shahid Beheshti University of Medical Sciences.

### 2.1 Sample Size Calculation

According to the article by Mohebi et al. the sample size was estimated to be 30 mothers. However, to determine the precise sample size, a pilot study was conducted involving 15 mothers.

$$N=1+\frac{2{\left(Z_{\propto }+Z_{\beta }\right)}^{2}K}{{\left(\mathrm{In}\;\left(\frac{F\left(\rho \right)}{F\left(\rho .\right)}\right)\right)}^{2}(K-1)}$$

$$F(X)=\frac{1+\left(K-1\right)X}{1-X}$$

Based on these formulas and assuming *α* (type I test error) of 0.05, *β* (type II test error) of 0.8, and *ρ* = 0.8 (based on the pilot study), the sample size for the main study was calculated to be 49 individuals. Considering the potential dropout rates, the final sample size comprised 114 individuals.

### 2.2 Demographic Data Collection

Upon enrollment, comprehensive demographic information was collected through structured surveys. Data gathered included:

- **Maternal Age:** Recorded in years.
- **Maternal Education Level:** Categorized as primary, secondary, or university degree.
- **Child's Age:** Recorded in years and months.
- **Child's Gender:** Noted as male or female.

This demographic information was analyzed to assess potential correlations with the accuracy of maternal assessments in detecting ECC.

### 2.3 Maternal Training and Image Acquisition

Participating mothers received standardized training designed to equip them with the skills necessary for effective intraoral photography and caries detection. A 6‐min instructional video was disseminated electronically via a secure platform, accessible through email and messaging applications such as WhatsApp (WhatsApp LLC). The video provided detailed guidance on identifying visual indicators of dental caries, including white spots and enamel demineralization, and offered step‐by‐step instructions for capturing high‐quality intraoral images.

Mothers were instructed to take five intraoral photographs of their child's teeth, ensuring coverage of occlusal, buccal, and lingual surfaces. Photographs were to be taken under natural lighting conditions with the smartphone's flash disabled to prevent glare, maintaining a consistent distance and angle to achieve clear and focused images. Captured images were uploaded to a secure, encrypted online platform compliant with data privacy regulations. In instances where images were deemed unclear or insufficient, mothers were contacted with feedback and requested to retake and resubmit the photographs.

### 2.4 Maternal Assessments

Following the successful upload of images, mothers conducted independent evaluations of the photographs using a simplified diagnostic checklist. This checklist guided mothers in identifying and documenting the presence of potential carious lesions on specific teeth. They were asked to report on their children's dental health status, including the presence/absence of caries and the number of caries‐affected teeth if present. The process aimed to empower mothers by actively involving them in the early detection of dental caries, fostering increased awareness and engagement in their children's oral health care.

### 2.5 Professional Evaluations

The images submitted by mothers were independently reviewed by two board‐certified pediatric dentists (PDs) who were blinded to the maternal assessments to eliminate bias. Each tooth in the images was classified into one of the following categories:

1. No Caries Present: Healthy tooth structure without signs of decay.
2. Early‐Stage Caries: Presence of white spots or enamel demineralization indicative of initial decay.
3. Cavitation Caries: Visible cavitation or breakdown of the tooth surface.

To establish a reference standard, all children also underwent an in‐person clinical examination conducted by a third independent dentist examiner (DE), who adhered to the World Health Organization (WHO) criteria for caries detection (World Health Organization 2013). Subsequently, they determined and recorded the presence or absence of dental caries and the number of carious teeth, if detected, based on photographs and clinical examinations. In cases where the two PDs' assessments differed, a senior PD provided an adjudicative review to reach a consensus diagnosis.

### 2.6 Handling Missing Data

To address missing data in our study, we employed multiple imputation techniques. This approach allowed us to estimate plausible values for missing entries, thereby preserving the dataset's integrity and ensuring robust statistical analyses. By incorporating multiple imputation, we minimized potential biases associated with missing information, leading to more reliable and valid conclusions.

### 2.7 Statistical Analysis

The accuracy of maternal assessments was evaluated by calculating sensitivity, specificity, positive predictive value (PPV), and negative predictive value (NPV) in comparison to professional evaluations and the independent DE's clinical examination. Sensitivity measured the proportion of correctly identified caries cases, while specificity determined the proportion of correctly identified non‐caries cases. PPV represented the likelihood that a positive maternal assessment indicated true caries presence, whereas NPV measured the probability that a negative maternal assessment accurately reflected the absence of caries.

To assess agreement across the three screening methods: maternal assessments, remote PD evaluations, and in‐person dental examinations (DEs), where ICCs were employed. ICC quantifies the degree of reliability and consistency among raters, with values ranging from 0 to 1, where higher values indicate stronger agreement. An ICC above 0.75 was considered excellent reliability, while values between 0.60 and 0.74 were classified as good agreement. This metric was chosen over traditional *κ* statistics to better capture variability across multiple raters and provide a more comprehensive assessment of diagnostic reliability. ICC analysis was conducted using a two‐way mixed‐effects model, assessing both absolute agreement and consistency among raters (Trevethan 2017).

All statistical analyses were conducted using IBM SPSS Statistics for Windows, Version 27.0 (IBM Corp 2024). Descriptive statistics were used to summarize demographic characteristics, and inferential tests were applied to assess diagnostic performance. A significance threshold of *p* < 0.05 was set to determine statistical significance. Additionally, 95% confidence intervals (CIs) were calculated to evaluate the precision of sensitivity, specificity, and agreement estimates.

## 3 Results

### 3.1 Participant Demographics

The study included 114 mother–child pairs, with children aged between 3 and 6 years. The mean age of the children was 4.8 ± 0.9 years, with the majority (64.0%) falling within the 4–6‐year age range (Table 1). Most children were 5 years old (30.7%), followed closely by 6‐year‐olds (33.3%), 4‐year‐olds (26.3%), and a smaller proportion of 3‐year‐olds (9.6%). The study comprised 64 boys (56.1%) and 50 girls (43.9%) (Table 1). Mothers had a mean age of 33.2 ± 5.6 years, with 42 mothers (36.8%) being 33 years old and younger and 37 mothers (32.5%) falling between 33 and 43 years of age. Regarding maternal education, 28 mothers (24.6%) had not completed high school, 42 (36.8%) had a high school diploma, and 21 (18.4%) held a university degree (Table 1). Maternal age and education information were not available for all participants, as reflected in Table 1. Among the 114 participating mothers, 58 (50.9%) successfully captured and submitted the required intraoral photographs after following the instructional video (Table 2).

**Table 1:** Demographic characteristics of study participants. Demographic distribution of the study, including child gender, age, maternal education level, and maternal age.

Variable	Category	*n* (%)
Child's sex	Male	64 (56.1%)
	Female	50 (43.9%)
Child's age (years)	3	11 (9.6%)
	4	30 (26.3%)
	5	35 (30.7%)
	6	38 (33.3%)
Mother's education	No high school diploma	28 (24.6%)
	High school diploma	42 (36.8%)
	University degree	21 (18.4%)
Mother's age (years)	< 33	42 (36.8%)
	33–43	37 (32.5%)

*Note:* Maternal age and education data were not available for all participants.

**Table 2:** Agreement and diagnostic performance of caries detection methods.

Diagnosis method	Teeth examined	Sensitivity (%)	Specificity (%)	False positives (%)	False negatives (%)	ICC with DE	ICC (all 3 methods)	*p* value
Maternal evaluation (ME)	58	100	100	0	0	0.909	0.978	< 0.0001
Pediatric dental expert (PDE)	58	100	100	0	0	0.988	0.978	< 0.0001
Dental examination (DE)	58	Reference	Reference	Reference	Reference	1.000	0.978	< 0.0001

*Note:* This table presents the diagnostic performance metrics—sensitivity, specificity, false positives, false negatives, and intraclass correlation coefficients (ICCs)—for maternal evaluations (MEs) and pediatric dental expert (PDE) assessments, using dentist examinations (DEs) as the reference standard. Both ME and PDE demonstrated perfect sensitivity and specificity (100%), with no false positives or negatives. The ICC values indicate a high degree of agreement with DE, suggesting that maternal assessments, when properly trained, can be as reliable as professional evaluations in detecting carious lesions.

### 3.2 Comparative Analysis of Caries Detection Methods

The accuracy of maternal evaluations (MEs) in detecting carious teeth was compared to assessments by pediatric dental experts (PDEs) and professional DEs. All comparative analyses were conducted among the 58 mother–child pairs who successfully submitted intraoral photographs. Mothers identified at least one carious tooth in 49 out of 58 cases (84.5%), while PDE identified caries in 51 out of 58 cases (87.9%). According to the DE, only 1 out of 20 cases (5%) exhibited at least one carious tooth, indicating discrepancies in detection rates among the different assessors. Sensitivity and specificity calculations demonstrated 100% sensitivity and 100% specificity for maternal assessments, indicating perfect agreement in identifying carious versus non‐carious teeth when compared to professional evaluations. These findings suggest that trained mothers can perform highly accurate screenings when using photographic methods.

### 3.3 Inter‐Rater Reliability

The interclass correlation coefficient was used to assess the consistency of caries detection across the three diagnostic methods. The agreement between MEs and professional DEs was 0.909, while the agreement between PDE and DE was 0.988, both indicating near‐perfect reliability. When all three assessment methods (ME, PDE, and DE) were considered together, the interclass correlation coefficient was 0.978, confirming a statistically significant level of agreement (*p* < 0.0001). These results validate the use of maternal‐assisted screening as an effective method when integrated into tele‐dentistry frameworks.

## 4 Discussion

ECC is a global public health concern, disproportionately affecting children in low‐resource settings where geographic, socioeconomic, and infrastructural barriers delay access to pediatric dental care. Limited availability of dental specialists contributes to the progression of untreated caries, leading to higher treatment costs and preventable oral health complications. Tele‐dentistry has emerged as a promising solution, enabling remote caries detection and reducing reliance on in‐person examinations (Ghorbani et al. 2018; Estai et al 2020, 2022; de Almeida Cardinobu et al. 2017). This study assessed the feasibility and accuracy of maternal‐assisted smartphone‐based screening, demonstrating that trained mothers can effectively contribute to early caries detection, reducing healthcare burdens and improving early intervention.

Our study evaluated the reliability of maternal‐assisted smartphone‐based screening for ECC, incorporating structured parental training with ICC‐based validation. Prior research focused primarily on school‐based screenings or professional‐led image capture, overlooking the potential of home‐based parental screening (Estai et al. 2020; Purohit et al. 2017; Saied‐Moallemi et al. 2008; Estai et al. 2022; Haddad et al. 2015; Kravitz et al. 2016; Summerfelt 2011; Ashtiani et al. 2024; AlShaya et al. 2020; Sakr et al. 2020; Xiao et al. 2021; Azimi et al. 2023; Saini et al. 2025; Price et al. 2025). We found strong agreement between maternal assessments and professional dental evaluations, with both sensitivity and specificity reaching 100%. The statistically significant ICC (*p* < 0.0001) further confirmed the reliability of parent‐assisted screening. MEs identified at least one carious lesion in 49 out of 58 cases, closely aligning with professional dental exams. Only 1 out of 20 cases was misidentified, reinforcing the diagnostic precision of trained maternal assessments. These findings suggest that mothers, when properly trained, can accurately assess their child's oral health, facilitating earlier intervention and reducing unnecessary clinic visits.

### 4.1 Addressing Access Barriers Through Tele‐Dentistry

Socioeconomic disparities significantly impact access to pediatric dental care, particularly in low‐resource settings where families face geographic, financial, and infrastructural barriers that delay routine visits (Elamin et al. 2021). Studies indicate that children from low‐income backgrounds have higher rates of untreated caries, largely due to limited access to preventive dental services (Javadzadeh et al. 2023). Limited integration of oral health within PHC systems further exacerbates these disparities, preventing timely intervention (Ghorbani et al. 2018). Tele‐dentistry presents a cost‐effective, scalable solution to overcome these challenges, enabling early screening and timely intervention for children in underserved regions (Estai et al. 2020, 2022). Our findings suggest that maternal‐assisted tele‐dentistry may significantly enhance access to care by empowering parents to participate in screening efforts, reducing the burden of in‐person visits, and allowing earlier identification of dental issues before they progress into severe conditions (Price et al. 2025).

### 4.2 Economic Benefits of Early Caries Screening for Families and the Nation

Beyond its diagnostic accuracy, maternal‐assisted tele‐dentistry has significant economic implications for both families and national healthcare systems. Preventive dental care is far more cost‐effective than managing advanced caries, which often requires invasive procedures such as extractions, restorations, or hospital‐based treatments (Ghorbani et al. 2018). Studies indicate that early intervention through tele‐dentistry may reduce treatment costs by minimizing the need for complex restorative procedures (Ghai 2020; Mohebbi et al. 2017; de Almeida Cardinobu et al. 2017; Mohebbi et al. 2017). This is particularly important globally, as public healthcare systems are already burdened with managing a high prevalence of preventable conditions. Expanding tele‐dentistry as a cost‐effective strategy may help alleviate financial strain, improve resource allocation, and increase access to preventive dental care, particularly in underserved regions.

For families, early home‐based screenings reduce the financial burden of frequent dental visits, transportation costs, and missed workdays. For the nation, integrating tele‐dentistry into PHC frameworks could reduce national healthcare expenditures on preventable oral health conditions, ensuring more efficient resource allocation (Saied‐Moallemi et al. 2008; Estai et al. 2022). Countries that have implemented tele‐dentistry‐based models have reported reduced hospitalization rates for severe caries cases and improved population‐wide dental health outcomes, reinforcing the long‐term cost savings of preventive care (WhatsApp LLC; Trevethan 2017; Summerfelt 2011).

### 4.3 Maternal‐Assisted Screening as a Sustainable Preventive Model

This study demonstrated a high degree of agreement between maternal assessments and dentist evaluations, with sensitivity and specificity reaching 100%. Similar studies confirm that trained caregivers can reliably contribute to early caries detection, reinforcing the feasibility of parent‐assisted screening models (Estai et al. 2020a; Xiao et al. 2021). Moreover, the ICC between MEs and dentist assessments (*p* < 0.0001) further validated the reliability of this approach (AlShaya et al. 2020). By incorporating structured oral health education into maternal training programs, tele‐dentistry can empower parents to play an active role in their child's dental health, reducing unnecessary referrals and promoting a culture of early prevention (Mohebbi et al. 2017; Azimi et al. 2023; Price et al. 2025).

### 4.4 Challenges and Implementation Considerations

Despite its advantages, maternal‐assisted tele‐dentistry presents challenges. Variability in smartphone access among mothers was noted, requiring alternative communication channels such as social media applications and email for data transmission (Purohit et al. 2017). Inconsistencies in image quality posed technical difficulties, highlighting the need for standardized training in dental photography (Sakr et al. 2025). Addressing these constraints through structured educational programs and digital literacy initiatives will be critical in ensuring equitable access to tele‐dentistry services (Azimi et al. 2023; Ashtiani et al. 2024).

This study has different limitations. First, maternal‐assisted assessments were based on visual evaluation of caries without distinguishing lesion activity or using diagnostic indices such as ICDAS. The selected age range of 3–6 years may also limit generalizability, though it was chosen given the higher accuracy of visual detection reported in this group. In addition, family income data were not collected, and the sample included a relatively high proportion of mothers with secondary or higher education, which may have influenced the results. Finally, diagnostic accuracy analyses were performed among the 58 mother–child pairs who completed both the photographic submission and evaluation process. These factors should be considered when interpreting the findings, and future studies should include more diverse populations and broader socioeconomic data.

### 4.5 Future Directions

Future research should evaluate the long‐term impact of tele‐dentistry on ECC prevention and treatment outcomes. Larger, multi‐center trials are needed to assess the scalability of this approach and its integration within national healthcare strategies (Kravitz et al. 2016; Saini et al. 2025). Further investigations into caregiver adherence, usability concerns, and barriers to widespread adoption will be essential in refining tele‐dentistry models for broader implementation (Xiao et al. 2021). Factors such as maternal oral health literacy and family income data should be considered in future studies. Ultimately, this study underscores the potential of maternal‐assisted tele‐dentistry as a cost‐effective, scalable, and sustainable model for advancing pediatric oral health (Azimi et al. 2023).

## Author Contributions

Parastoo Iranparvar and Zahra Ghorbani planned and conducted the study, collected data, and assisted in manuscript preparation. Sajjad Zandieh and Serlie Hartoonian collected data and assisted in manuscript preparation. Mohsen Shirazi supervised the study and contributed to all steps, including study design, data collection, analysis, and manuscript writing. All authors reviewed and approved the final manuscript.

## Conflicts of Interest

The authors declare no conflicts of interest.

## Data Availability

The datasets generated and analyzed during the current study are not publicly available due to patient privacy and ethical restrictions. De‐identified data may be available from the corresponding author upon reasonable request and with appropriate institutional approvals.
